# Patellar Base Support Technique During Manipulation Under Anesthesia for Knee Arthrofibrosis Limits the Risk of Iatrogenic Complications

**DOI:** 10.1016/j.eats.2023.08.001

**Published:** 2023-11-27

**Authors:** Konrad Malinowski, Marcin Mostowy, Michał Kanak, Przemysław A. Pękala, Dong Woon Kim, Nicholas I. Kennedy, Robert F. LaPrade

**Affiliations:** a Department of Anatomy, Jagiellonian University Medical College, International Evidence-Based Anatomy Working Group, Kraków, Poland; b Artromedical Orthopedic Clinic, Bełchatów, Poland; c Orthopedic and Trauma Department, Veterans Memorial Teaching Hospital in Lodz, Medical University of Lodz, Lodz, Poland; d Faculty of Medicine and Health Sciences, Andrzej Frycz Modrzewski Kraków University, Kraków, Poland; e Lesser Poland Orthopedic and Rehabilitation Hospital, Kraków, Poland; f Twin Cities Orthopedics, Edina, Minnesota, U.S.A.

## Abstract

Knee extension contracture is a common postinjury and postsurgical complication, which decreases knee joint flexion. Many techniques have been described in the literature to restore knee flexion, with the most common one being an arthroscopic lysis of adhesions. However, in severe cases, additional intra- and extra-articular procedures are needed to restore full knee flexion. Manipulation under anesthesia (MUA) is one of them. Unfortunately, it may lead to devastating complications, such as iatrogenic rupture of the patellar tendon or fractures of the patella or tibial tuberosity. Therefore, the purpose of this report is to present a safer modification of MUA for knee extension contracture in cases in which excessive force is demanded to achieve flexion. The key aim of the “patellar base support” technique (PBS technique) is to stretch the contracted quadriceps muscle with controlled and decreased tension on the patella, patellar tendon, and tibial tuberosity.

## Introduction

Knee extension contracture is a common postinjury and post-surgical complication, which decreases knee joint flexion.^1^, ^2^, ^3^, ^4^, ^5^ Knee flexion loss due to an extension contracture may arise both from intra-articular adhesions and extra-articular reasons. Among extra-articular origins, the most common ones are quadriceps tendon contracture, limitation of gliding between quadriceps muscle parts and surrounding tissues, and generalized stiffness or fibrosis of the extensor apparatus.^1^ When physiotherapy fails, surgical management is conducted.

Arthroscopic lysis of adhesions (LOA) is considered to be an effective surgical solution for intra-articular contracture origins.^1^^,^^6^ Unsatisfactory ROM after a LOA gives rise to the need of additional procedures. One of the most widespread is manipulation under anesthesia (MUA), which may result in immediate gain of ROM.^6^, ^7^, ^8^ MUA may be also performed as an isolated procedure without a LOA, i.e., in cases of isolated extra-articular contracture or shortly after the initial procedure, to avoid opening the joint once again.^8^ However, this procedure is not free of possible devastating complications, like iatrogenic rupture of the patellar tendon, fracture of the patella or tibial tuberosity or patellofemoral chondral damage.^1^^,^^2^ Therefore, the aim of this report is to present a safer modification of MUA for knee extension contracture—a “patellar base support” technique (PBS technique). In cases when excessive force would be demanded to achieve flexion, this technique could be used. The key aim of the procedure is to stretch the contracted quadriceps muscle with controlled and decreased tension on the patella, patellar tendon, and tibial tuberosity.

## Surgical technique

### Indications

This surgical technique is indicated for knee extension contracture not sufficiently responding to the arthroscopic LOA, quadriceps tendon contracture, limitation of gliding between quadriceps muscle parts and surrounding tissues, stiffness or fibrosis of extension apparatus, and decreased the bone quality of the patella.

### Contraindications

The contraindications for using this technique are recent extension apparatus tears, recent patellar fractures, and active knee intra- and peri-articular infections.

### Patient Positioning

The technique is performed in patients positioned supine under general or spinal anesthesia. After tourniquet placement, the affected leg is prepared and draped in a sterile fashion.

### Procedure

The technique may be performed with or without arthroscopic assistance.

### Preparation to PBS Technique Without Arthroscopic Assistance

**Fig 1:**
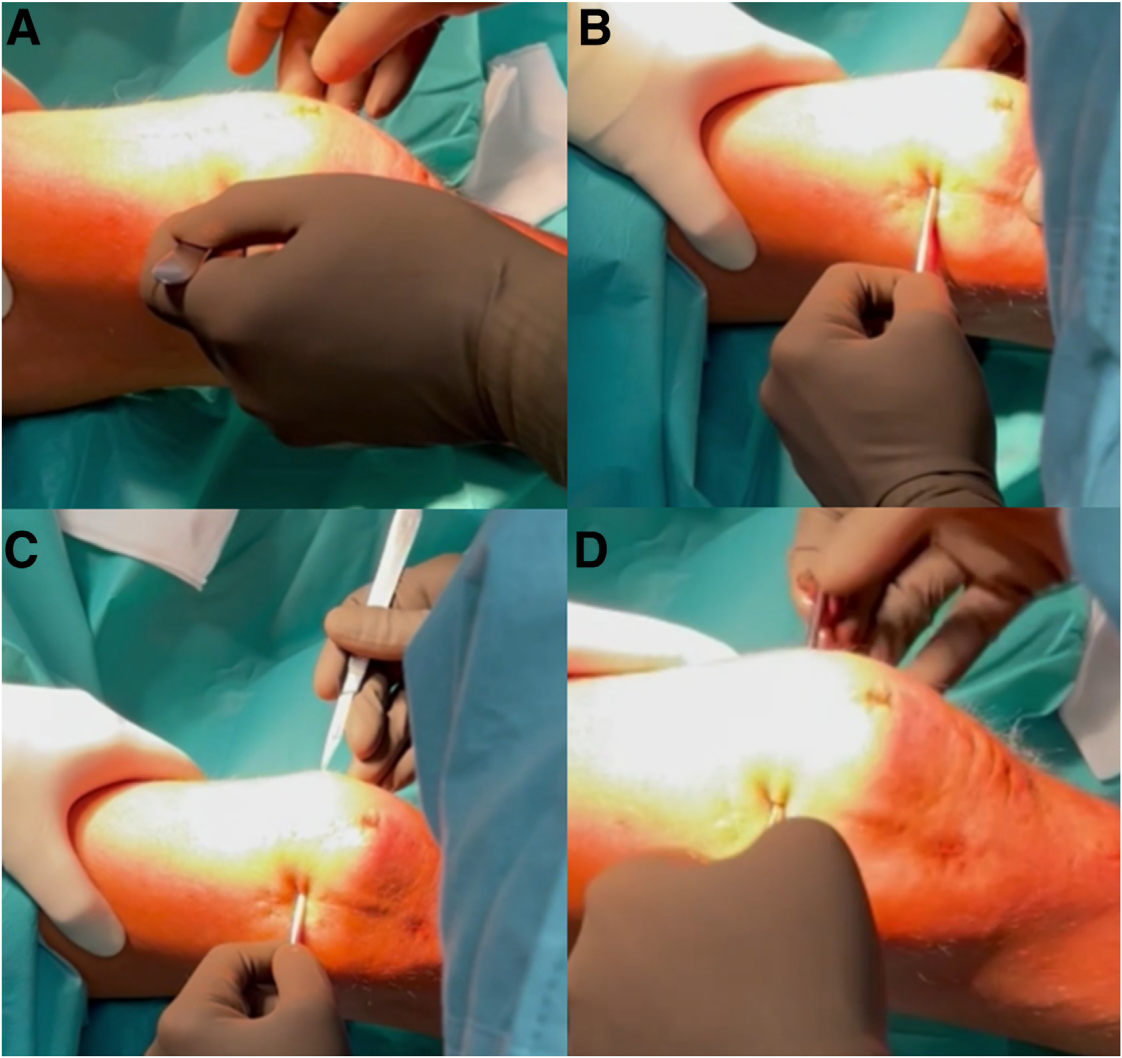
Preparation for “patellar base support” (PBS) technique without arthroscopic assistance. The right knee is shown with the patient supine. (A) Creation of superolateral approach. (B) Introduction of the switching stick into the suprapatellar recess. (C) Creation of superomedial approach. (D) Switching stick is pulled out over the skin.

A standard superolateral portal (in the close relation to the superolateral edge of the patellar base) is made with a stab incision using a no. 11 blade that reaches the suprapatellar recess (Fig 1A and Video 1). Then, a switching-stick (ConMed, Warsaw, Poland) is introduced through the portal to enter the suprapatellar recess (Fig 1B, Video 1). The switching-stick is pressed anteriorly and distally against patellar base and pushed medially, until palpable under the skin on its usual location of superomedial portal. The skin is incised over the palpable instrument; thus, the superomedial portal is made in inside-out manner (Fig 1C, Video 1). The switching stick is then pulled out over the skin (Fig 1D, Video 1).

### Preparation to PBS Technique With Arthroscopic Assistance

**Fig 2:**
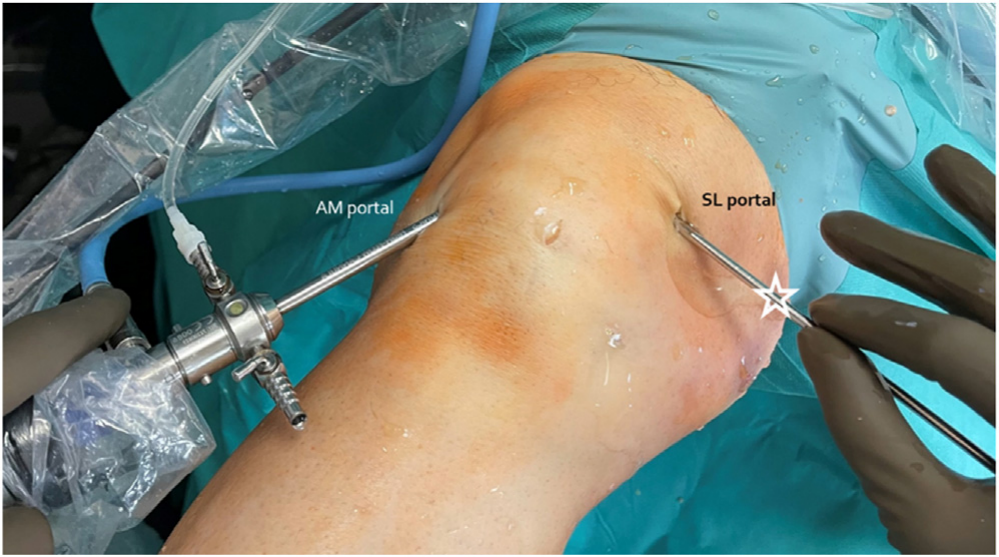
Preparation for patellar base support PBS technique with arthroscopic assistance. The left knee is shown with the patient supine. White asterisk denotes the switching stick. AM, anteromedial; SL, superolateral.

**Fig 3:**
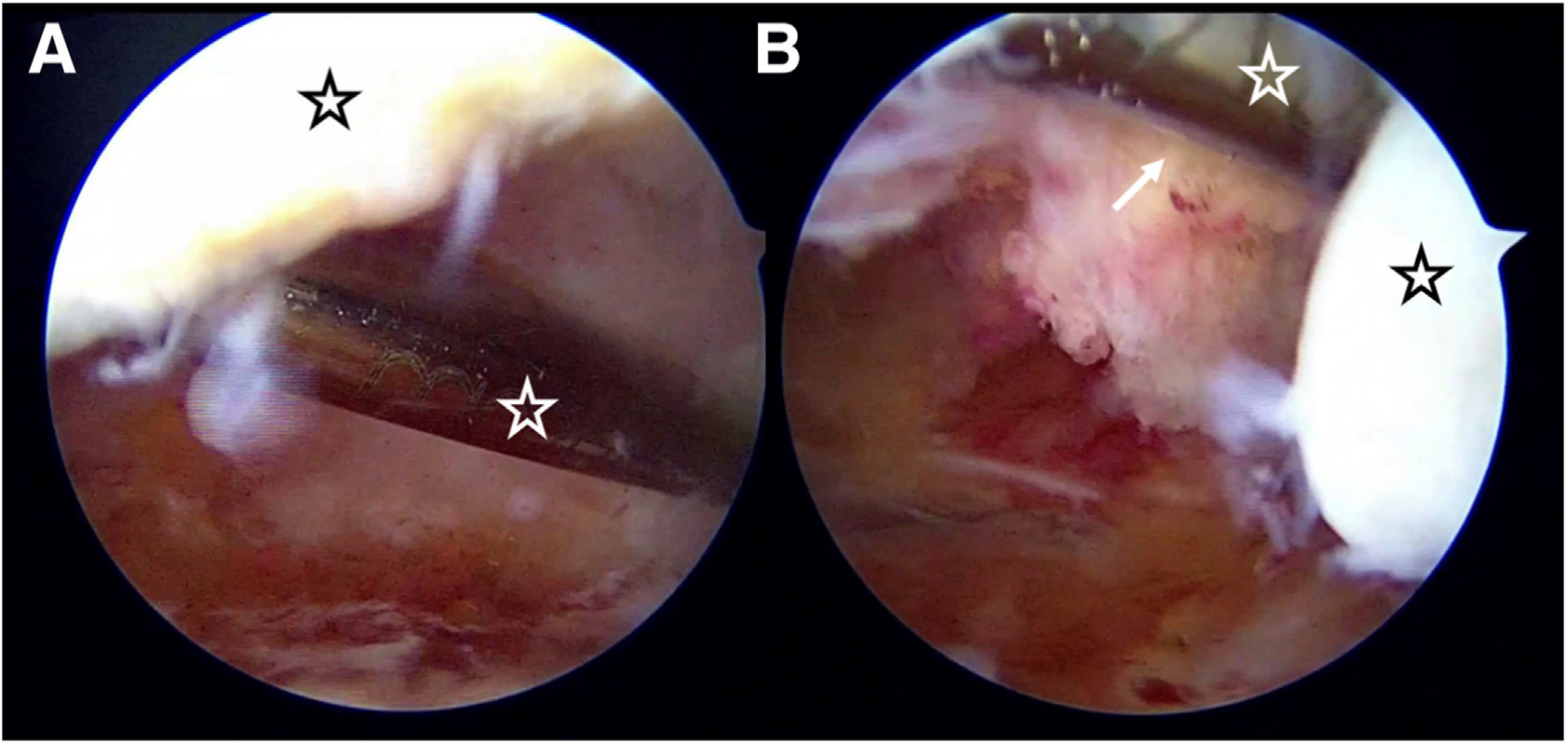
Introduction and stabilization of the switching stick on the patellar base. The left knee is shown with the patient supine. (A) Introduction of the switching stick into the suprapatellar recess. (B) Stabilization of the switching-stick on the patellar base. White asterisks denote to the switching stick; black asterisks denote the patella; white arrow denotes the distal part of quadriceps tendon merged with suprapatellar fat pad.

**Fig 4:**
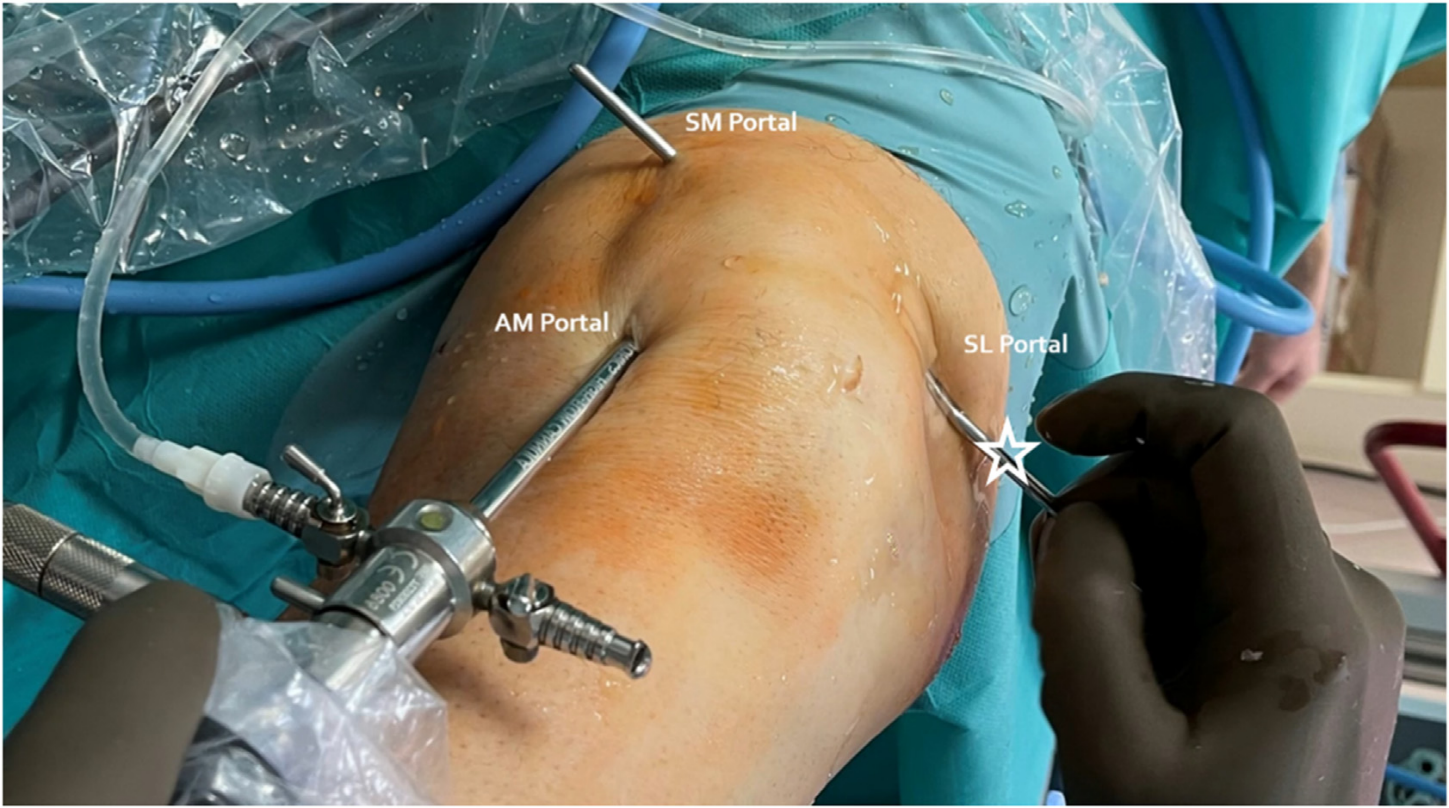
Switching stick pulled out over the skin after arthroscopic preparation to the PBS technique. The left knee is shown with the patient supine. White asterisk denotes the switching stick. AM, anteromedial; SL, superolateral; SM, superomedial.

The arthroscope (ConMed, Warsaw, Poland) is introduced through a standard anteromedial portal. After visualizing the lateral part of the suprapatellar recess at the level of the patellar base, a standard superolateral portal is made. Under visual control, the switching stick is introduced into the suprapatellar recess (Figs 2 and 3A, Video 1). The switching stick is placed and stabilized on the patellar base adjacent to the distal enthesis of the quadriceps muscle (Fig 3B, Video 1). Then, it is pushed medially until palpable under the skin. After creating the superomedial portal over palpable instrument in the inside-out manner with the stab incision, the switching-stick is pulled out over the skin (Fig 4, Video 1).

### PBS Technique

**Fig 5:**
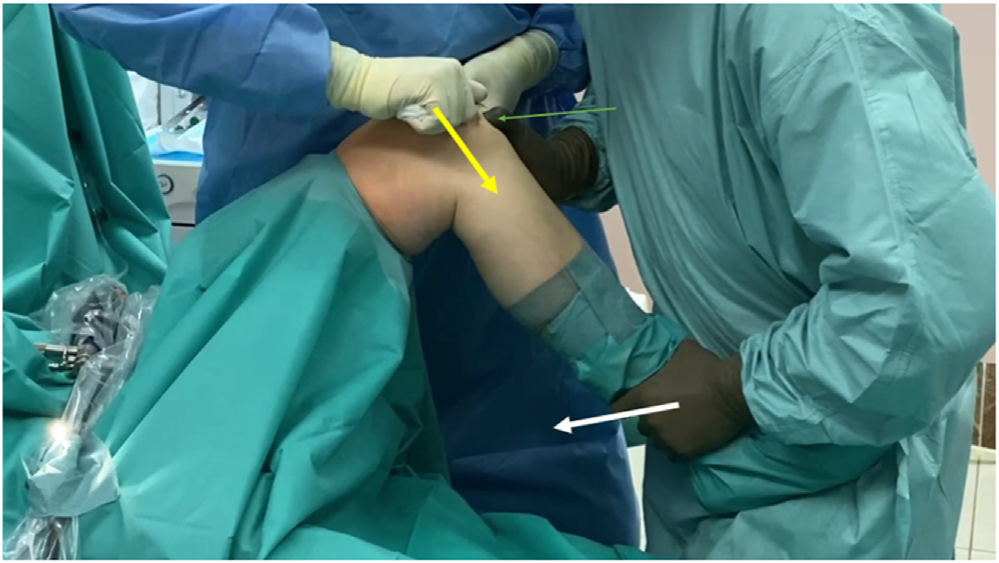
Patellar base support technique with marked force vectors, side view. The left knee is shown with patient supine. Yellow arrow denotes the assistant pushes the stick distally and anteriorly with simultaneous stabilization of the switching stick on patellar base; green arrow denotes the main surgeon controls the tension of the patellar tendon with his/her thumb; and white arrow denotes using the other hand the main surgeon bends the knee into maximal flexion.

**Fig 6:**
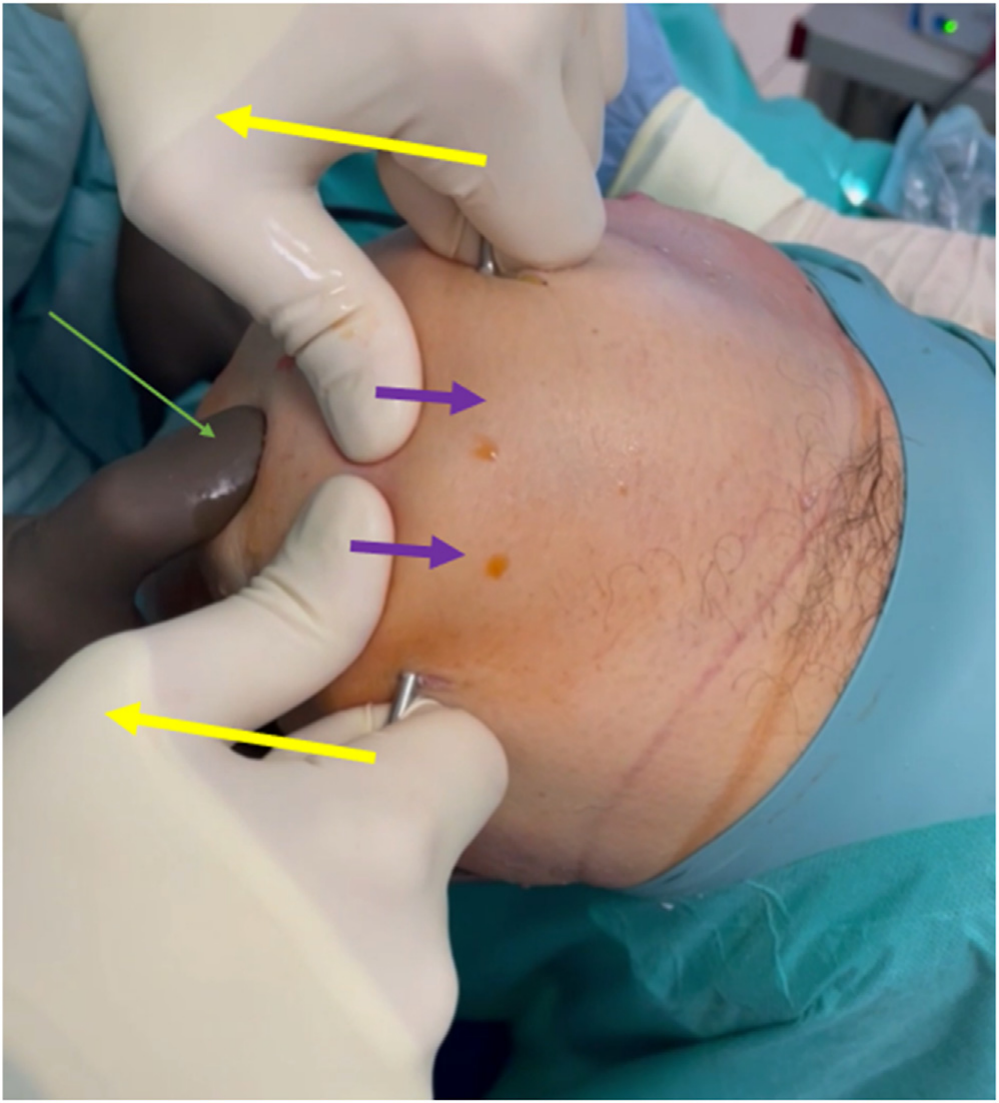
Patellar base support technique with marked force vectors, superior view. The left knee is shown with patient supine. Yellow arrows shows the assistant pulling the stick distally and anteriorly; purple arrows show the assistant simultaneously stabilizing the switching stick on the patellar base; the green arrow shows the main surgeon controlling the tension of the patellar tendon with his/her thumb.

The next step is common for the technique with or without arthroscopic assistance. Proper stabilization of the switching stick on the base of the patella is achieved as follows: the assistant pushes the stick distally and anteriorly with simultaneous stabilization of the patella by the force exerted proximally and posteriorly. When the switching stick is securely “locked”, the assistant exerts distal force on the patellar base by the handle, thus, created (Fig 5, Video 1). In the meantime, the main surgeon controls the tension of the patellar tendon with his/her thumb—it should decrease after the assistant introduces distal force to the patellar base. Using the other hand, the main surgeon bends the knee into maximal flexion, uninterruptedly controlling the tension of the patellar tendon (Fig 5, Video 1). In such a way, the main surgeon can be sure that the force is mainly exerted on the quadriceps muscle. The distal force to the patellar base can be introduced by pushing or pulling (Figs 5 and 6, Video 1), according to the surgeon and assistant preference.

### Rehabilitation

During the first 2 postoperative weeks, the patient spends 5 minutes every 2 hours sitting with the knee gravitationally hyperextended and spends another 5 minutes with the joint flexed maximally until the presentation of pain. Ambulation using crutches with increasing weightbearing is allowed, dependent on knee tolerance. Guided physiotherapy administered weekly is prescribed after the first postoperative week.

## Discussion

The main indication for the presented PBS technique is knee extension contracture, in which an isolated LOA did not result in knee flexion restoration, and therefore, excessive force would be demanded to achieve flexion. By the means of patellar base support, this modification of a MUA allows the surgeon to stretch the contracted quadriceps muscle in a safer manner with controlled and decreased tension on the patella, patellar tendon and tibial tuberosity, as well as on the patellofemoral cartilage.

### PBS Technique as a Way to Avoid Iatrogenic Rupture of Patellar Tendon, Fracture of Patella or Tibial Tuberosity

Decreased tension on the patella, patellar tendon, and tibial tuberosity is very important, as traditional MUA is not free of possible devastating complications. The most common ones are patellar fractures, tibial tuberosity fractures and patellar tendon tears.^2^^,^^8^ On the other hand, in the literature, we only managed to find one report of a quadriceps tendon tear, as a result of MUA.^8^ Therefore, the presented PBS technique allows to “transfer” the force from the more vulnerable parts of the extension mechanism (patella, patellar tendon, and tibial tuberosity) to the more resistant quadriceps muscle-tendon unit and its patellar insertion. The quadriceps muscle-tendon unit is composed of both muscular and tendinous tissue, which makes it inherently more prone to elongation. Although in a given patient, the tendinous portion of the quadriceps muscle-tendon unit may not be very prone to stretching, its muscular “red” part should be able to stretch, even in presence of fibrosis.^9^, ^10^, ^11^ Also, the quadriceps tendon is much longer than the patellar tendon. Different studies have reported a mean length of the patellar tendon to be about 45-52 mm in length, while the quadriceps muscle-tendon unit may very well be over 40 cm in length.^12^, ^13^, ^14^, ^15^ Therefore, even if we theoretically assume similar percentages of possible elongation, the longer structure would elongate more. The quadriceps tendon was also reported to have higher ultimate loads than the patellar tendon.^12^

### PBS technique as a Way to Avoid Patellofemoral Cartilage Overload During MUA

**Fig 7:**
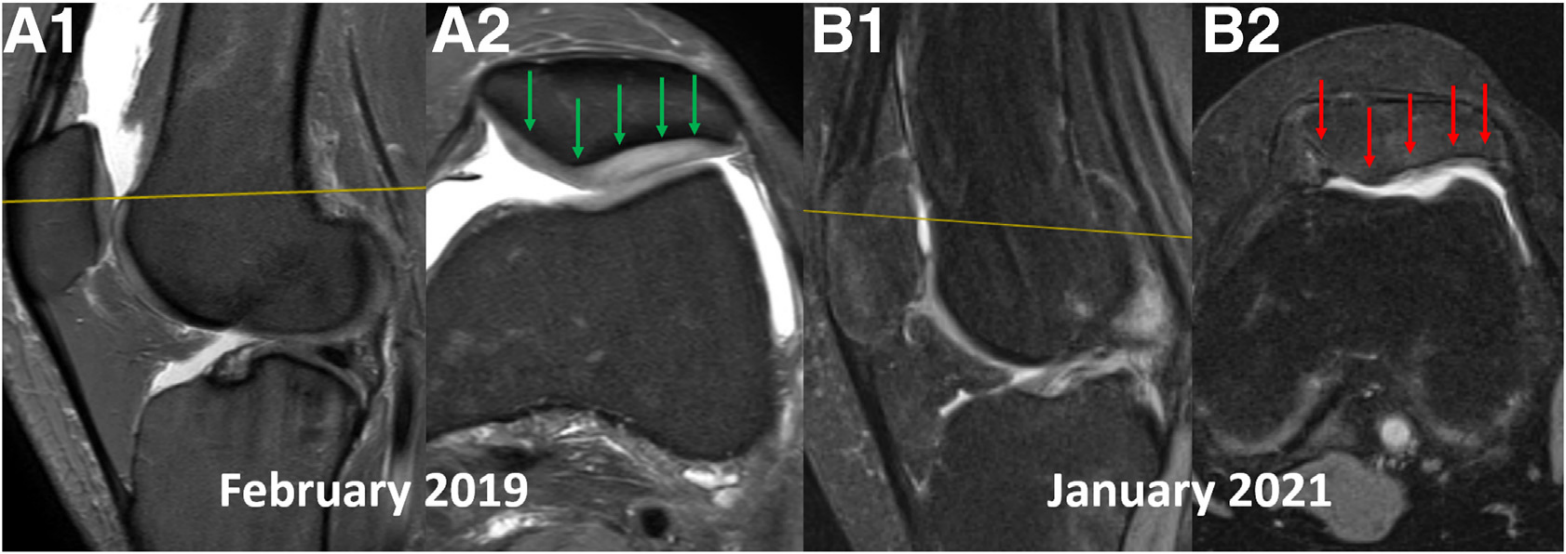
Severe patellofemoral cartilage damage in a relatively short time in a patient with knee extension contracture, due to overload during rehabilitation by means of painful, excessively forceful flexion. The left knee, sagittal (A1 and B1) and axial (A2 and B2) MRI scans. The patient, who has a knee extension contracture, was rehabilitated by means of painful, excessively forceful flexion. While the improvement in knee flexion was minimal, the patellofemoral cartilage (arrows) was severely damaged in a relatively short time (February 2019 to January 2021) as a result of these manipulations.

Besides described intraoperative complications, there are also high concerns as to consequences of manipulation on the patellofemoral cartilage.^1^^,^^2^ Such negative consequences of excessive overload on patellofemoral cartilage may potentially be avoided during a MUA by the means of PBS technique (Fig 7).

### Comparison of PBS Technique With Similar Techniques Described in the Literature

Shen et al. described a surgical technique with similar idea to the PBS technique presented in this article; however, it encompassed longer times of direct quadriceps stretching (usually more than 10 days) and was more invasive.^16^ The prolonged patellar traction was achieved through the Kirschner wires introduced in the patella. Shen et al. described it on a group of patients with a stiff knee after femoral fractures and treated with modified Judet’s quadricepsplasty.^16^ However, the authors reported complications, such as patellar fractures, ischemia, and necrosis of the prepatellar skin, and intolerable pain.^16^ Despite those disadvantages, longer stretching of quadriceps muscle complex may be useful in severe cases of generalized stiffness or fibrosis of extension apparatus. The presented PBS technique is designed for cases in which isolated arthroscopic LOA is not enough to restore knee flexion; however, extension apparatus may still be prone to controlled, direct stretching.

### Proposed Place of PBS Technique in the Knee Extension Treatment Workflow

**Fig 8:**
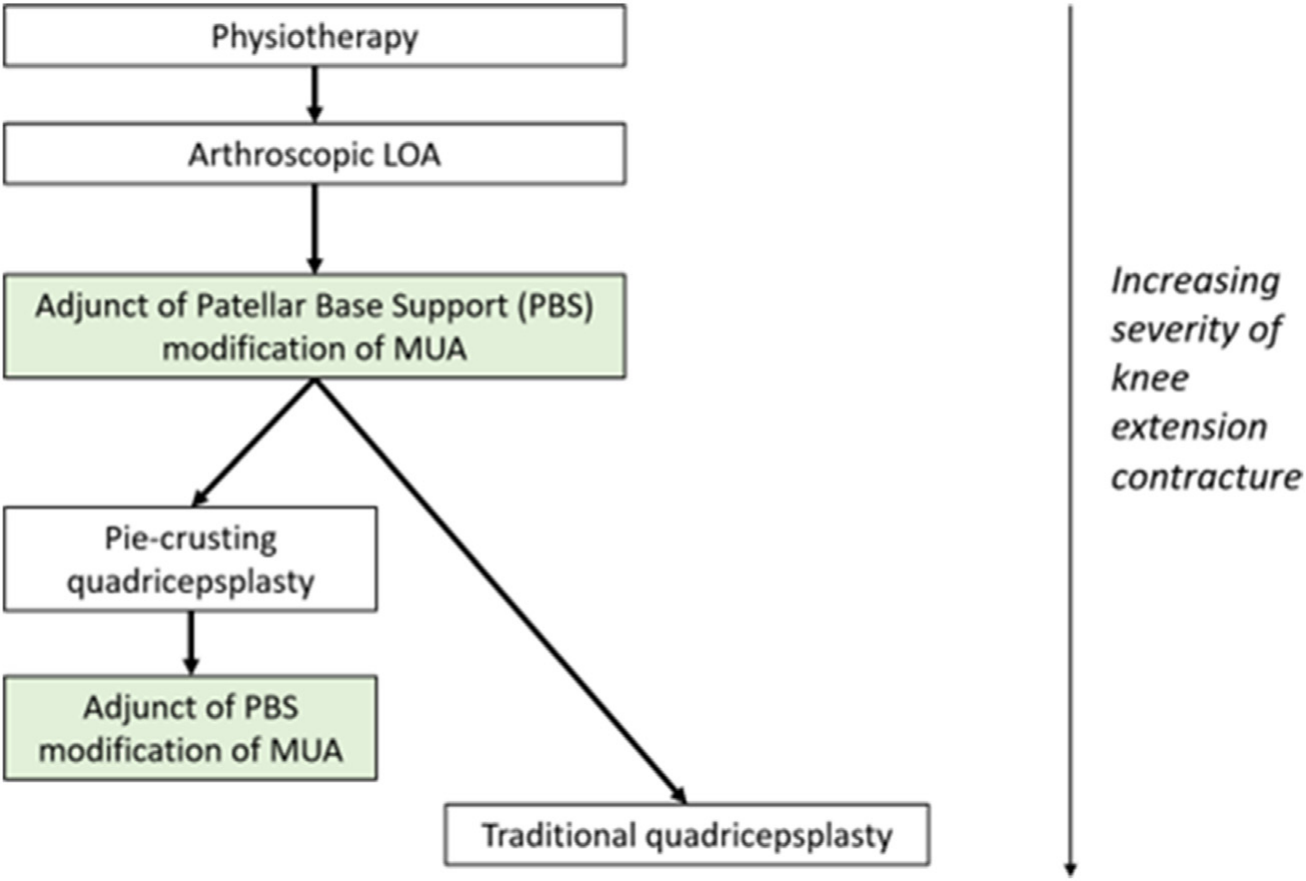
Proposed workflow for knee extension contracture treatment. LOA, lysis of adhesions; MUA, manipulation under anesthesia; PBS, patellar base support.

Nowadays, arthroscopic lysis of adhesions (LOA) is the most commonly performed procedure and is considered as a standard technique for the surgical treatment of a stiff knee (Fig 8), due to its less-invasive nature in comparison to open procedures.^1^^,^^2^^,^^6^ According to the literature, one of the indications for MUA is as an additional procedure after LOA to break hidden adhesions (Fig 8).^1^^,^^2^ Such hidden adhesions may present both intra-articularly and between quadriceps muscle parts and surrounding tissues.^1^^,^^2^^,^^17^ However, if the extension contracture persists after arthroscopic LOA, the quadriceps is usually the main remaining source of knee flexion deficit, which is confirmed by effectiveness of quadricepsplasty in such cases.^1^

Therefore, as the next step after failure of LOA, we propose to modify the traditional MUA into the PBS technique, which allows the contracted quadriceps to stretch directly and its surrounding tissues and to break up hidden extra-articular adhesions (Fig 8).^1^^,^^2^ In severe flexion deficit, quadricepsplasty is a viable option (Fig 8). In less severe cases, the PBS technique may allow it to achieve the flexion in a safer manner than a traditional MUA. As described above, it may allow one to diminish the risk of injury to the patella, patellar tendon, tibial tuberosity, or patellofemoral cartilage. The PBS technique may also provide a next surgical step before the necessity of quadricepsplasty. Therefore, possible complications of traditional quadricepsplasties demanding excessive exposure (such as Judet’s or Thompson’s) may be avoided.^1^^,^^8^^,^^16^^,^^18^^,^^19^ However, in the recent years, less invasive modification of quadricepsplasty, so-called pie-crusting quadricepsplasty was developed.^10^^,^^11^^,^^17^ This procedure is routinely finished with MUA in order to stretch the prepared quadriceps. This is the next step in proposed workflow for knee extension contracture treatment, where PBS technique may be used instead of a traditional MUA (Fig 8).

To summarize, the PBS technique is a safer and cost-effective modification of MUA for knee extension contracture, in cases in which excessive force would be demanded to achieve flexion. This technique allows the contracted quadriceps muscle to be directly stretched with decreased tension on the patellar tendon, which potentially decreases the risk of iatrogenic rupture of the patellar tendon, fracture of the patella or tibial tuberosity, or patellofemoral chondral damage. It may be useful as an adjunct technique in each case of MUA application, i.e., after arthroscopic LOA or pie-crusting quadricepsplasty. The advantages and disadvantages of the technique are summarized in the Table 1.

**Table 1:** Advantages and Disadvantages of “Lever on Quadriceps” Technique

Advantages	Disadvantages
Decreased tension on patellar tendon, which potentially decreases the risk of injury to the patella, patellofemoral cartilage, patellar tendon, and tibial tuberosity Possibility of uninterruptedly controlling the decreased tension of the patellar tendon Only basic instruments needed Cost-effective Simple and easy-to-learn technique May be performed with or without arthroscopic assistance May serve an adjunct technique in each case of MUA application, i.e., after arthroscopic LOA or pie-crusting quadricepsplasty Less invasive than the techniques using longer time of patellar traction	Two surgeons are necessary. Additional portals in suprapatellar region Only one-time stretching of quadriceps is possible, as opposed to the techniques that use a longer time for patellar traction Risk of typical MUA complications when performed improperly Frequent kinesiotherapy (passive flexion every hour for first 2 weeks) is recommended.

LOA, lysis of adhesions; MUA, manipulation under anesthesia.

## Supplementary Data

ICMJE author disclosure forms

Video 1 Patellar base support (PBS) technique, a modification of manipulation under anesthesia (MUA), is presented. Its aim is to directly stretch the contracted quadriceps muscle during manipulation for knee extension contracture to diminish the risk of iatrogenic rupture of the extensor mechanism. The technique may be performed with or without arthroscopic assistance. The required approaches are standard superolateral and superomedial. After the creation of the superolateral approach, a switching-stick is introduced into the suprapatellar recess and pressed onto the skin, marking a spot for superomedial approach. Afterward, the switching-stick is pulled out over the skin. The switching-stick is stabilized on the patellar base. The arthroscopically assisted PBS technique requires the same approaches. We can see the switching-stick introduced through the superolateral portal into the suprapatellar recess from the outside and arthroscopically. The switching-stick is then stabilized on the patellar base near the distal part of quadriceps tendon and suprapatellar fat pad. Afterward, it is pulled out over the skin. After either nonarthroscopic or arthroscopic preparation, the PBS technique is begun. The assistant pushes the stick distally and anteriorly, while the main surgeon controls the tension of the patellar tendon with his/her thumb, using the other hand to bend the knee into maximal flexion. According to the surgeon’s preference, the assistant may also exert the force on the patella by pulling instead of pushing. It is important to properly lock and stabilize the switching stick on the patellar base before the force is introduced.
